# *QuickStats:* Percentage* of Families That Often or Sometimes Did Not Have Enough Food To Last 30 Days and Did Not Have Enough Money to Buy More,**^†^** by Poverty Status**^§^** — National Health Interview Survey, United States, 2018

**DOI:** 10.15585/mmwr.mm6926a5

**Published:** 2020-07-03

**Figure:**
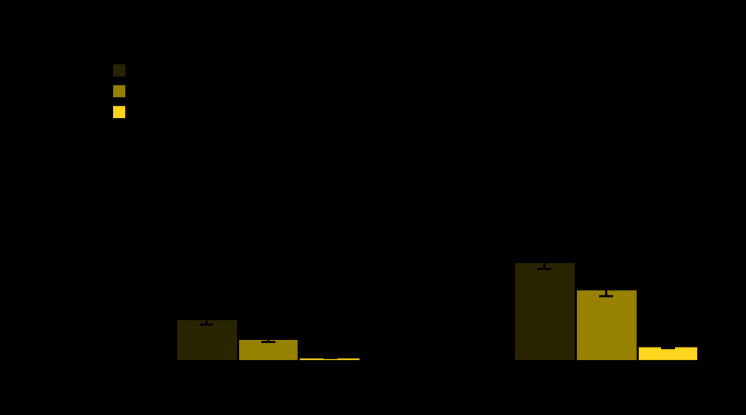
During 2018, 2.7% of U.S. families often did not have enough food and did not have enough money to buy more to last 30 days. Poor families (9.6%) were more likely than near-poor families (4.9%) and not-poor families (0.8%) to often lack food. An estimated 8.2% of families sometimes did not have enough food or the money to buy more, and the percentage varied by poverty status: poor families (22.6%), near-poor families (16.2%), and not-poor families (3.4%).

**Source:** National Health Interview Survey, 2018 data. https://www.cdc.gov/nchs/nhis.htm.

